# Synthesis, characterization, and evaluation of Hesperetin nanocrystals for regenerative dentistry

**DOI:** 10.1038/s41598-023-28267-y

**Published:** 2023-02-06

**Authors:** Mahdieh Alipour, Simin Sharifi, Mohammad Samiei, Shahriar Shahi, Marziyeh Aghazadeh, Solmaz Maleki Dizaj

**Affiliations:** 1 Dental and Periodontal Research Center, Faculty of Dentistry, Tabriz University of Medical Sciences, Daneshgah St, Golgasht St, Tabriz, Iran; 2 Department of Endodontics, Faculty of Dentistry, Tabriz University of Medical Sciences, Tabriz, Iran; 3 Stem Cell Research Center, Tabriz University of Medical Sciences, Tabriz, Iran; 4 Department of Oral Medicine, Faculty of Dentistry, Tabriz University of Medical Sciences, Daneshgah St, Golgasht St, Tabriz, Iran

**Keywords:** Stem cells, Nanoscience and technology

## Abstract

Hesperetin (HS), a metabolite of hesperidin, is a polyphenolic component of citrus fruits. This ingredient has a potential role in bone strength and the osteogenic differentiation. The bone loss in the orofacial region may occur due to the inflammation response of host tissues. Nanotechnology applications have been harshly entered the field of regenerative medicine to improve the efficacy of the materials and substances. In the current study, the hesperetin nanocrystals were synthesized and characterized. Then, the anti-inflammatory and antioxidative effects of these nanocrystals were evaluated on inflamed human Dental Pulp Stem Cells (hDPSCs) and monocytes (U937). Moreover, the osteoinduction capacity of these nanocrystals was assessed by gene and protein expression levels of osteogenic specific markers including RUNX2, ALP, OCN, Col1a1, and BSP in hDPSCs. The deposition of calcium nodules in the presence of hesperetin and hesperetin nanocrystals was also assessed. The results revealed the successful fabrication of hesperetin nanocrystals with an average size of 100 nm. The levels of TNF, IL6, and reactive oxygen species (ROS) in inflamed hDPSCs and U937 significantly decreased in the presence of hesperetin nanocrystals. Furthermore, these nanocrystals induced osteogenic differentiation in hDPSCs. These results demonstrated the positive and effective role of fabricated nanocrystal forms of this natural ingredient for regenerative medicine purposes.

## Introduction

Bone loss and resorption in the oral cavity are challenging due to the inflammatory nature of these phenomena. This inflammation happens because of the complex environment of the oral cavity, which contains a wide range of microorganisms. Any connections between the oral cavity and bone tissue through inflamed or exposed pulp tissue or trauma may cause local bone resorption^1^. Moreover, in oral infectious situations such as periodontitis bacterial by-products suppress the host tissue response and its native regenerative capacity by recruitment of inflammatory cells^2,3^. The inflammatory response in the oral cavity interferes with the natural healing and regeneration of bone tissue in this area^4^. Therefore, the suitable substance for bone regeneration in this area should provide osteogenic capacities as well as anti-inflammatory characteristics.

Natural ingredients in the human diet, such as flavonoids with polyphenolic groups, affect bone metabolism and strength^5–7^. Hesperetin (3′,5,7-trihydroxy-4-methoxyflavanone) is a metabolite of hesperidin, a flavanone subgroup of flavonoids^7,8^. This natural ingredient indicates a wide range of biological activities such as antioxidative and anti-inflammatory effects as well as other flavonoids^6,9–13^. Moreover, hesperetin is an accessible and cost–benefit natural ingredient suggested as a bioactive substance for bone and Cartilage repair^7,14^.

Especially in abnormal systemic circumstances such as type I diabetes, it has been shown that hesperetin is able to suppress the high glucose inhibited osteogenesis by anti-inflammatory and antioxidative capacities^8^.

Nanomedicine has been introduced in regenerative medicine as a promising approach for elevating the characteristics of therapeutic substances. In most situations, the fabrication of nanoscale particles of different ingredients can improve their properties^15^.

Human dental pulp stem cells (hDPSCs) are one of the available sources of mesenchymal stem cells, which are able to differentiate into various cell types. Their availability and multi-differentiation capacity make these cells a promising source of stem cells for regenerative dentistry. Although these cells indicate significant proliferation and differentiation capacities, the presence of a signaling molecule that can induce osteogenesis in these cells is essential^16–19^. Different studies indicated the comparable capacity of hDPSCs in bone tissue engineering with bone marrow mesenchymal stem cells^20–22^.

In the current study, the authors hypothesized that by fabricating nanoscale hesperetin particles, they are able to improve this substance's characteristics for regenerative purposes. Therefore, hesperetin nanoparticles were synthesized for the first time, and their proliferative, anti-inflammatory, antioxidative, and osteogenic induction capacities were evaluated.

## Results and discussion

### Synthesis, characterization, and evaluation of hesperetin nanocrystals

#### Particle size

**Figure 1:**
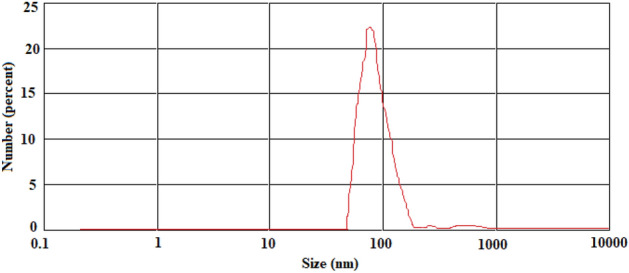
The mean diameter of hesperetin nanoparticles.

Various characteristics of nanoparticles in biomedical applications are influenced by particle size. Regarding the particle size distribution, PDI values more than 0.7 indicate the highly extensive distribution of the particle size; its analysis by the DLS method may probably be inappropriate^23^. Figure 1 depicts the size distribution of the formulated nanomaterials.

According to the results of DLS, an average size of 100 nm (PDI of 0.33) was demonstrated, which shows the production of mono-dispersed nanoparticles^24,25^.

### Scanning electron microscopy (SEM)

**Figure 2:**
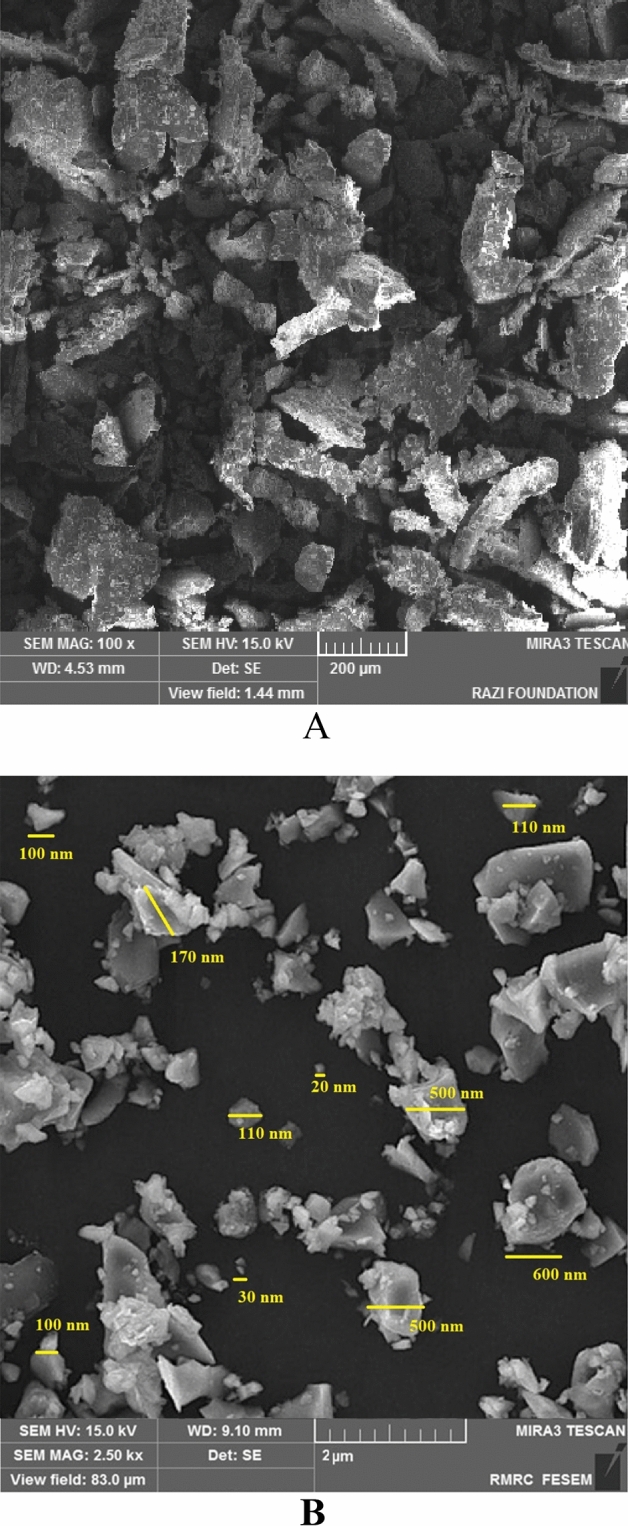
SEM image of (**A**) Hesperetin powder, (**B**) Hesperetin nanoparticles.

In the SEM image, the nanoparticles were detectable as irregular polygonal in shape. SEM evaluation demonstrated the size distribution of hesperetin powder (Fig. 2A) and hesperetin nanoparticles (Fig. 2B). The mean ± SD of the synthesized nanoparticles was 263.75 ± 214.18 nm.

Our results showed that the nanoparticle sizes differed in DLS analysis from SEM analysis. This may be due to hydrating the outer layer of the nanoparticles in the DLS method^25^. Sousa et al. described the same results previously^26^. Furthermore, aggregation of nanoparticles and non spherical shape of nanoparticles could be the reason for this difference. The synthesized nanoparticles have different dimensions, and they are flake-like (having a different aspect ratio compared to a sphere), which can cause this difference^27–29^.

#### Surface charge

**Figure 3:**
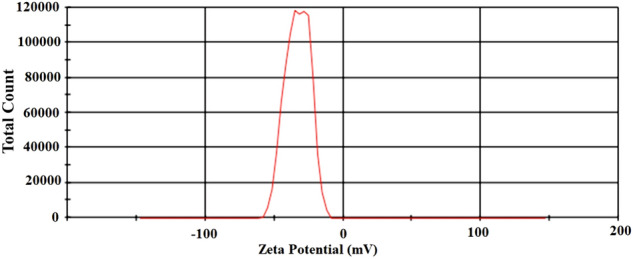
Zeta potential (ζ) distribution of nanoparticles.

A comparison was presented between the zeta potential (ζ) distribution of nanoparticles and the entire quantity; the corresponding measurement was − 32.9 ± 1.5 Mv (Fig. 3). The zeta potential with extreme negativity showed that the colloidal form of the nanoparticles was stable^30^.

#### Crystalline state

**Figure 4:**
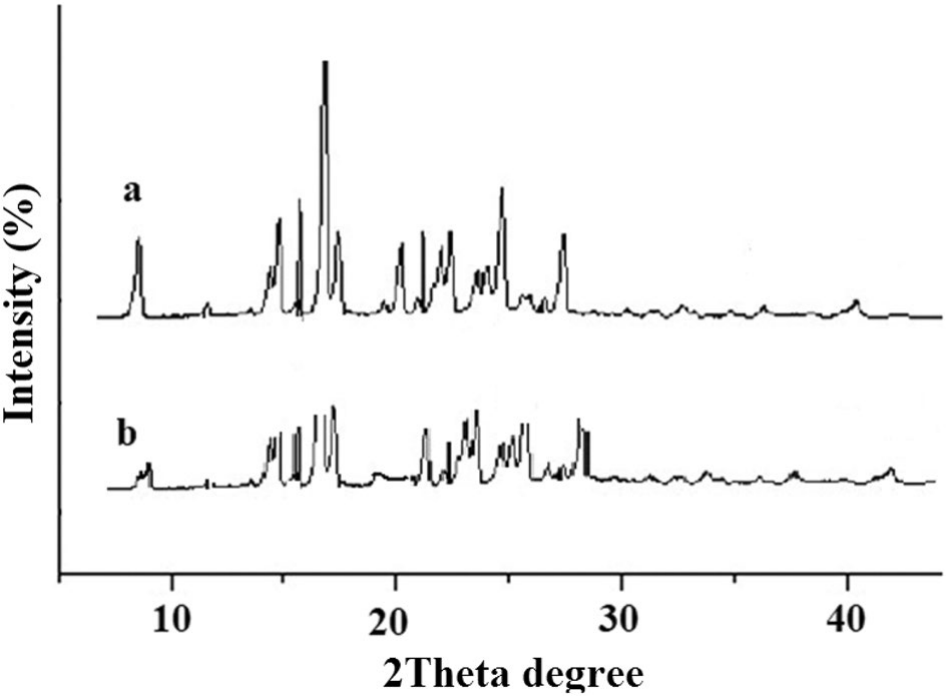
The X-ray diffractions of (**a**) free hesperetin and (**b**) hesperetin nanoparticles.

The associated X-ray diffraction models of free hesperetin and nanoparticles of hesperetin are presented in Fig. 4. Both of them demonstrated the distinctive crystalline peaks of 2θ, including values of 8.21°, 14.39°, 17.23°, 21.10°, 24°, 25.90°, and 29.68°^25^. The prepared nanoparticles showed lower X-ray diffraction than the free hesperetin, signifying the lower crystallinity and the smaller sizes^28^.

#### The molecular structure

**Figure 5:**
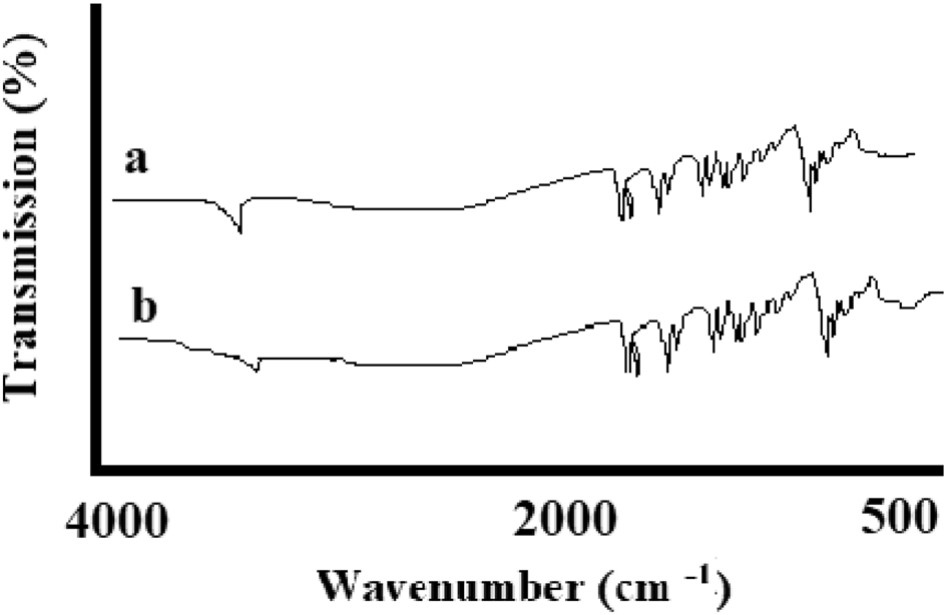
The FT-IR peaks of free hesperetin (**a**) and hesperetin nanoparticles (**b**).

FT-IR demonstrated free hesperetin and nanoparticles of hesperetin, and it was noted that the locations of the bands were not accompanied by any changes (Fig. 5). Two bands attributed to the operational array of the combined ether of C-O when saturated and aromatic at respective rates of 1040 cm^−1^ and 1260 cm^−1^ were demonstrated by the peaks. Moreover, aromatic diverse stretching of C=C between 1500 and 1580 cm^−1^, carbonyl group (C=O) band at 1635 cm^−1^, –OH stretching vibration at 3495 cm^−1^ associated with the bonding of hydrogen atom at an intermolecular level became recognized for hesperetin^25^. Other researchers have formerly described the identical FT-IR ranges^24^. It could be stated that spray drying did not alternate the chemical compositions of hesperetin.

#### MTT assay

**Figure 6:**
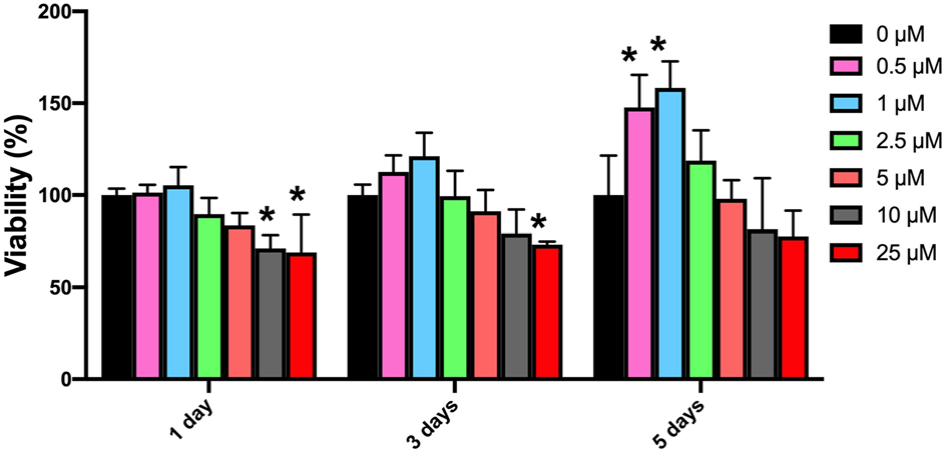
Comparative diagram of MTT test for evaluating different concentrations of nano-hesperetin on hDPSCs after 1, 3, and 5 days. In this assay the black column (0 µl) was considered as free substance group and contained cells alone.

Nanomedicine and the application of nanoparticles have been increased due to the significant and enhanced effects of these materials^31^. The fabrication and introduction of new nanoscale bioactive molecules with improved characteristics for inducing regeneration in mesenchymal stem cells provided a promising alternative^15,18,31^. Hesperetin is proposed as the anti-cancer agent that decreases cancer cell proliferation. The study by Yang et al. suggested that 100 μM of this substance reduced the proliferation of MDA-MB-231 breast cancer cell lines^32^. However, as well as other flavonoids, this bioactive material represents proliferative effects on mesenchymal stem cells in low concentrations^31^. Xue et al. suggested that hesperetin in low concentrations promotes the proliferation of human mesenchymal stem cells^7^. In the current study, to evaluate the proliferative concentration of nano-hesperetin on human dental pulp stem cells, various concentrations of this substance were added to each well while the control group was considered as cells alone, without any substances. The results of the MTT test after 1, 3, and 5 days were demonstrated in Fig. 6. These results indicated that not only nano-hesperetin did not have cytotoxic effects at 0.5 and 1 μM concentrations, but also it had positive proliferative effects on human dental pulp stem cells leading to significantly increasing of vital cells on day 5.

### Anti-inflammatory and antioxidative tests

**Figure 7:**
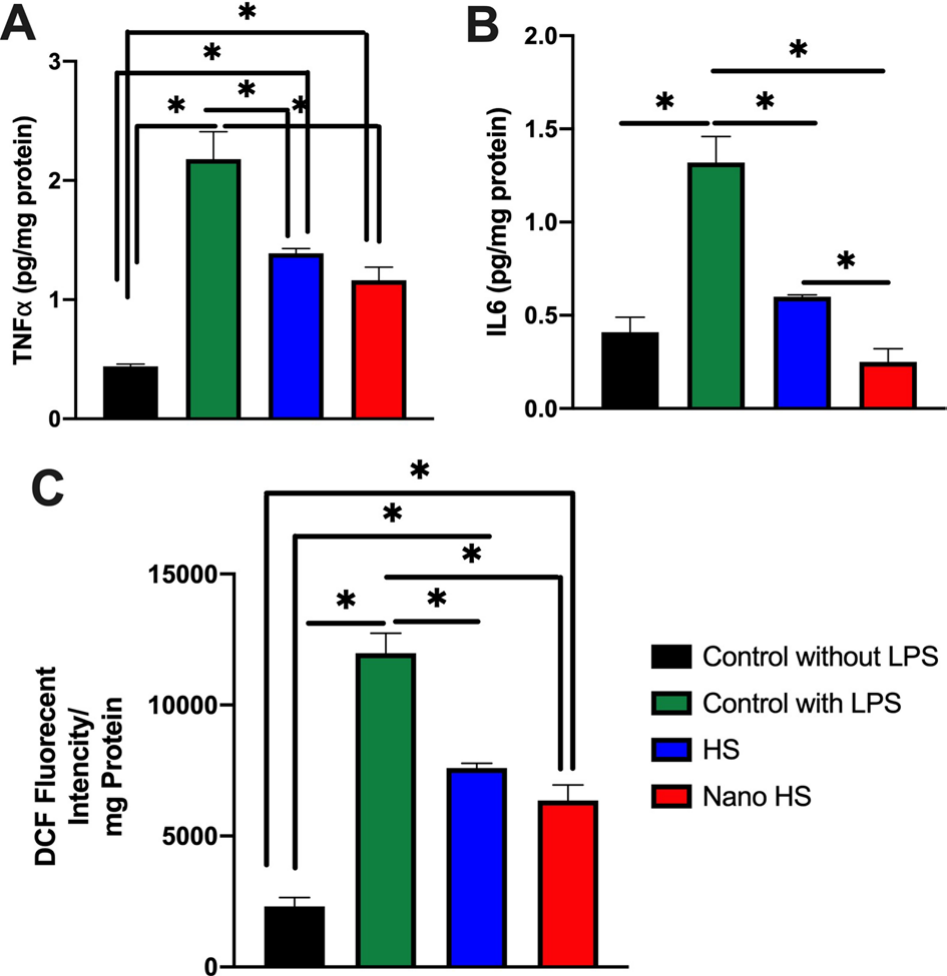
Measurement of intracellular inflammatory cytokines in LPS induced DPSCs. (**A**) The expression of TNF*α*; (**B**) the expression of IL6, and (**C**) DCF fluorescence intensity. *p < 0.05.

**Figure 8:**
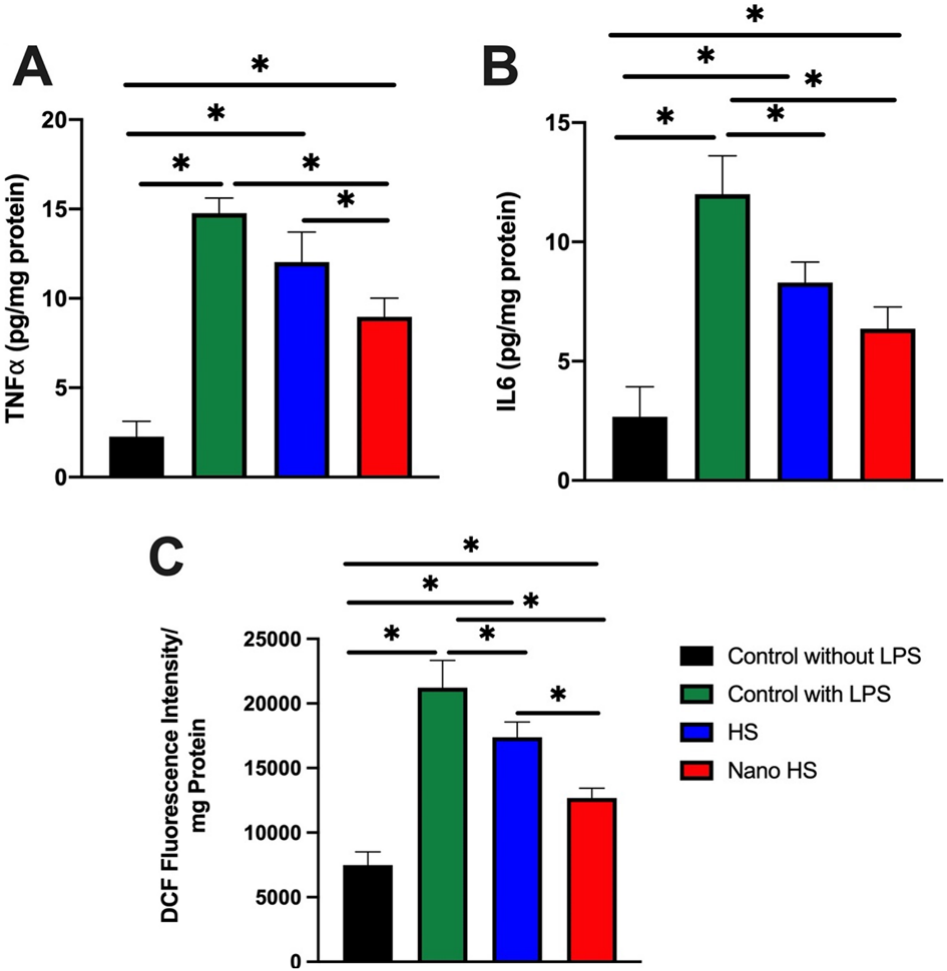
Measurement of intracellular inflammatory cytokines in LPS-induced U937. (**A**) The expression of TNF*α*; (**B**) the expression of IL6, and (**C**) DCF fluorescence intensity. *p < 0.05.

Hesperetin is known for its anti-inflammatory and antioxidative properties as well as other flavonoids^6,8,9,33^. Pinho-Ribeiro et al. evaluated hesperidin in the rodent model to decrease inflammation-associated pain. Their results showed that this substance reduces inflammatory pain by suppressing the inflammatory cytokine cascade, NF-kß pathway, and ROS activity^34^. Furthermore, applying hesperidin (glycoside form of the hesperetin) for skin injuries revealed the healing properties of this component. According to the results, hesperetin inhibited oxidative stress and inflammatory signals using down-regulation of critical cytokines in the inflammatory cascade, including TNF-, IL1ß, IL-6, and IL 10^35,36^. These interleukins and cytokines are widely involved in different inflammatory responses in the body, such as tooth and bone inflammation^6,9^. In the current study, human dental pulp stem cells were treated with both hesperetin and nano-hesperetin and inflamed by bacterial LPS and. The results of this test indicate that the nanoformulation of this natural substance is much more effective than hesperetin in decreasing the inflammatory response (Fig. 7). To evaluate the effect of hesperetin and nano-hesperetin on immune cells, which are known as main components in inflammation, the test was repeated on the human monocyte cell line U937 (Fig. 8). These cells were selected since they are appropriate in vitro models for the evaluation of inflammatory responses^37,38^.

### Alizarin Red S staining

**Figure 9:**
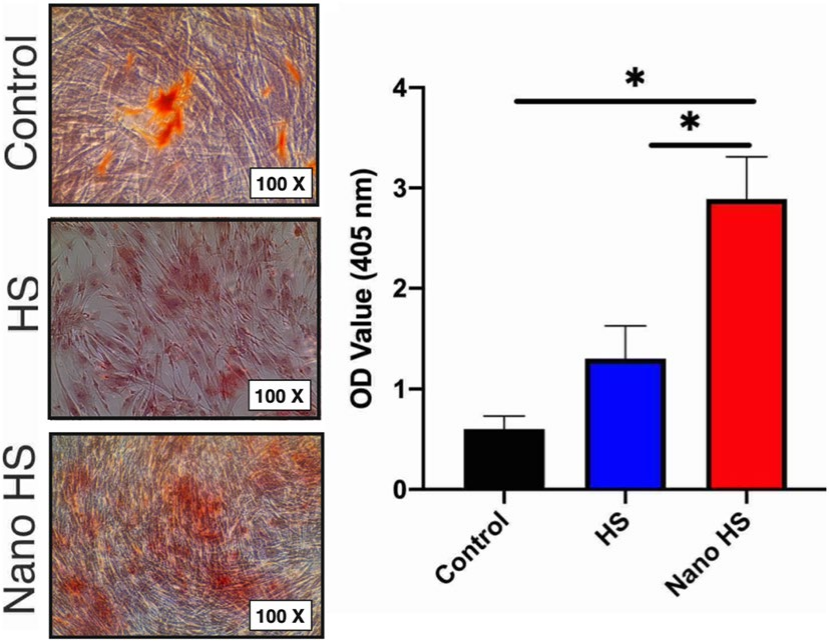
Qualitative and quantitative alizarin red analysis of hDPSCs cultured on a 24-well plate in the presence of hesperetin and nano-hesperetin particles and without substances (control). *p < 0.05.

Several pieces of evidence showed the stimulation of bone metabolism by flavonoids led to increased bone mineralization^39,40^. As a member of this family, hesperetin presented an increased level of mineralization in various studies^41^. However, due to the application of nanoparticles and improvement of biomaterials’ properties by synthesizing on the nanoscale, in the current study, the fabricated nano-hesperetin particles were added to the cell culture medium with a concentration of 1 μm. Three weeks after facing DPSCs cells to this biomaterial, the amount of mineralization was detected in each group to clarify the capacity of hesperetin particles in improving of mineralization. The results demonstrated higher mineralization induction in the nano-hesperetin group than in the hesperetin-treated and control (non-treated group) groups (Fig. 9). These results indicated a high capacity of hesperetin nanoparticles in induction of mineralization.

#### Real-time RT PCR and Western blot tests

There are several natural extracts representing bone formation by stimulating osteogenesis. Zhang et al*.* showed that flavonoids enhance osteogenic differentiation in hBMSCs^42^. Furthermore, Hokmabad et al. showed that the extract of Elaeagnus *Angustifolia*, a rich source of flavonoids, increased the expression of osteogenic genes^43^. Kim et al*.* evaluated the osteogenic effects of hesperetin on the differentiation of periodontal ligament stem cells and proved its osteogenic capacity^8^.

Trzeciakiewicz evaluated this flavonoid on primary rat osteoblasts cell lines and suggested that hesperetin influences osteoblast differentiation through the BMP signaling pathway. Moreover, their results demonstrated higher expression of ALP, Runx2, and Osterix in the hesperetin-treated group^41^.

**Figure 10:**
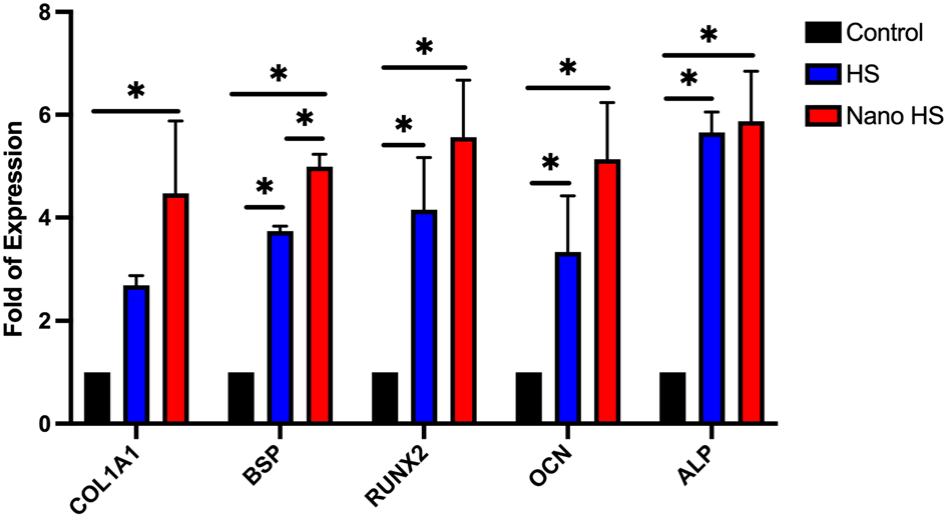
Expression levels of osteogenic genes in hDPSCs treated by hesperetin and nano-hesperetin particles. The results suggested that hesperetin in both forms significantly increased osteogenesis in hDPSCs (*p ≤ 0.05). Although nanoformulation of this natural ingredient showed elevated expression levels of genes compared to the HS group, the statistical difference was only reported in BSP. Control contained cells alone without substances.

**Figure 11:**
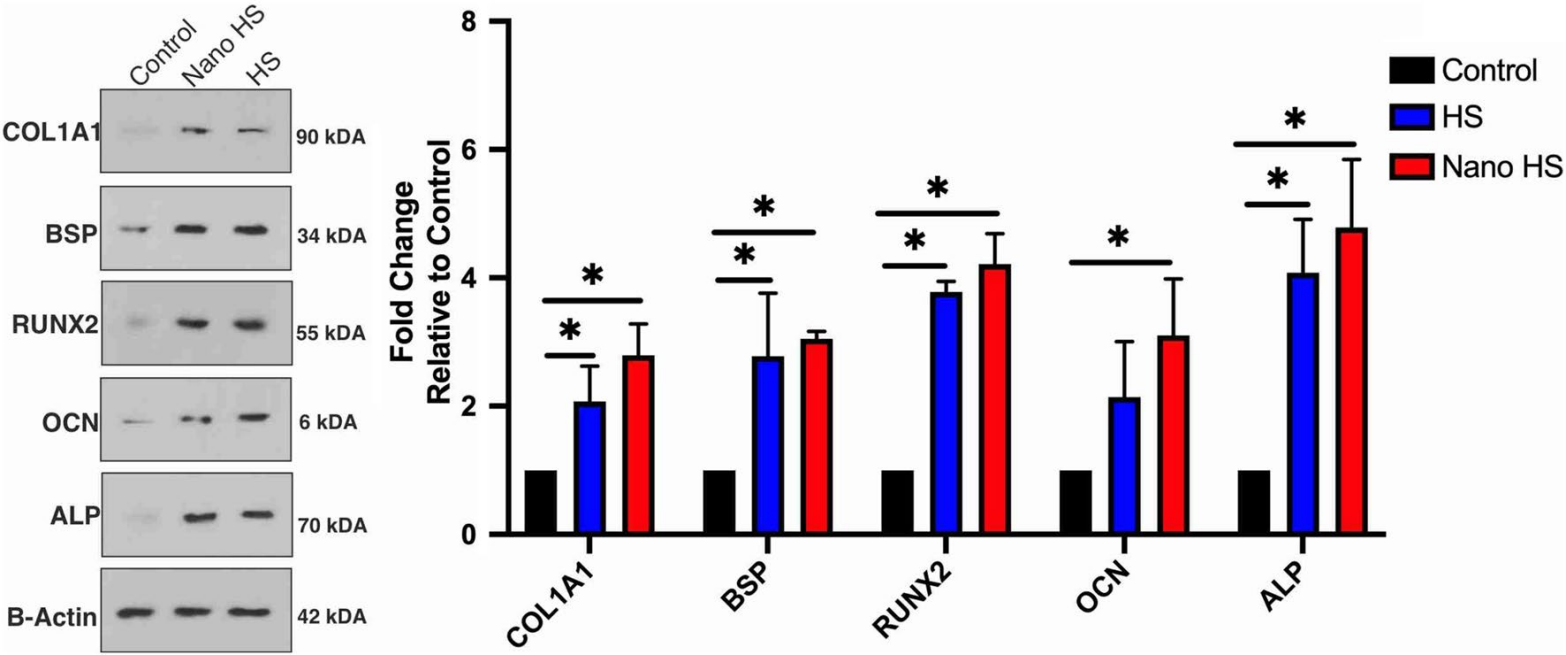
Immunoblotting assay for the proteins of osteogenic markers showed increased levels of these proteins in hDPSCs 21 days after exposure to the hesperetin and nano-hesperetin formulation. Control group presents the cells without substances (*p ≤ 0.05).

In the current study, the expression of five osteogenic markers, including Col1a1, BSP, Runx2, OCN, and ALP, was evaluated in gene and protein levels. The results are demonstrated in Figs. 10 and 11. The relative expression of Col1a1, BSP, Runx2, OCN, and Alp was 4.47, 4.98, 5.56, 5.13, and 5.87, respectively, in the nono-hesperetin group. These amounts were significantly higher compared to the control group. Nano- hesperetin particles elevated the expression of all evaluated genes compared to hesperetin group. This difference was significant in the expression of BSP.

The expression of the mentioned factors in protein levels were assessed and showed a remarkable increase in the nano-hesperetin group. These data were in a line with PCR results and supported the idea of improved characteristics of bioactive molecules on the nanoscale. Hence, it could be concluded that fabricated nano-hesperetin particles could improve the biological characteristics and enhance the osteogenic induction capacity of this flavonoid.

## Conclusion

The result of the current study suggested that nano-hesperetin has positive effects on osteogenic differentiation and proliferative effect on human dental pulp stem cells, nano-hesperetin can be considered an ideal option in bone and tooth tissue engineering. In addition, hesperetin nanoparticles can be applied to expand suitable scaffolds for bone, teeth, and periodontium regeneration.

## Materials and methods

### Synthesis, characterization, and evaluation of Hesperetin nanocrystals

Hesperetin was purchased from SIGMA-ALDRICH Co. (CAS: 69097-99-0. MW: 302.28 g/mol). Two mg of hesperetin was added to 20 ml of acetone on a stirrer (450 rpm). This solution was then drop-wised into a sodium dodecyl sulfonate (SDS) solution (0.001 M) under a homogenizer (10,000 rpm). The resulting solution was then placed in a spray-drier at a 2.5 ml/min rate at 30° C to produce hesperetin nanoparticles. Then we used dynamic light scattering (DLS) technique to measure the average diameter of nanoparticles using a Nano-ZS tool (Malvern Instruments Ltd., UK). Nanoparticles were sonicated before size identification (Power 500 W, 20% amplitude, 30 min of response time, pulse on 2 s, and pulse off: 2 s in an ice bath) at 25 ± 1 °C. Morphology identification of nanoparticles was conducted through SEM analysis via a scanning electron microscope (SEM, TESCAN, Warrendale, PA). To fix the nanoparticles in their dry powder form, an adhesive tape was used followed by applying gold coatings. The size of nano hespertin particles was determined from SEM micrographs using Image J software (NIH, USA, version 1.52n) as described previously^44^.

A zeta-sizer (Malvern, UK) was used to specify the surface charge of nano-scale particles according to the evaluation of zeta potential at 25 °C using a fresh aqueous suspension of nanoparticles that perfused into the capillary cell of the zeta-sizer. The process was followed by using X-ray diffraction (XRD) method to investigate the comparison of the crystalline state of the samples in a qualitative fashion (Philips TW 1710 diffractometer with Cu-Kα incident radiation adjusted at 40 kV and 30 mA). We chose room temperature to conduct the records over the 2θ range of 20°–60° at a scanning rate of 3˚/min.

Fourier transform infrared spectroscopy (FTIR) was utilized to obtain the structure of an inter/intramolecular bonding of the prepared nanomaterials (Termo Nicolet-6700, spectrophotometer) ranging from 4000 to 400 cm^−1^.

### Determination of proliferative concentration of hesperetin nanocrystals on hDPSCs by MTT assay

For evaluation of nano-hesperetin crystals’ effects on the proliferation and viability of hDPSCs, the MTT assay was performed. For this purpose, human dental pulp stem cells (hDPSCs) were obtained from the biobank of Shahid Beheshti University (Tehran, Iran). The cells were cultured in a culture medium containing DMEM high glucose (Gibco co.), 10% FBS (Gibco co.), and 1X pen/strep (Gibco co.) in a 75 cm^2^ culture flask. After obtaining 2 × 10^6^ cells, the cells were trypsinized and cultured in three 96 well plates (5000 cells/well) to evaluate the different concentrations of nano-hesperetin after 1, 3, and 5 days. The seeded cells were exposed to nano-hesperetin at concentrations of 0, 0.5, 1, 2.5, 5, 10, and 25 μM. After the treatment times, the medium was exchanged with 200 μl of MTT solution (0.5 mg/ml) and incubated for 4 h at 37 °C, away from light. Then the medium was replaced with 200 μl DMSO. Finally, the absorbance optical density was read by a plate reader (Awareness Technologies Stat Fax 2100 Microplate Reader) at 570 nm. The percentage of living cells was evaluated by comparing the control (cells grown in the absence of nano-hesperetin). All tests were performed in three replications, and data were reported as mean ± SD.

### Anti-inflammatory and antioxidative effects of hesperetin nanocrystals on inflamed U937

To evaluate the anti-inflammatory and antioxidative properties of hesperetin and hesperetin nanocrystals on inflamed hDPSCs, the antioxidative and anti-inflammatory tests were performed as mentioned previously^45^. Briefly, hDPSCs were cultured on 6 well plates (1 × 10^6^) and inflamed with 10 ng/ml LPS of E. Coli. (Sigma-Aldrich Co. Steinem, Germany). These plates were incubated for 24 h. To investigate the concentration of TNFᾳ and IL6 in the presence of nano hesperetin (1 μM) and hesperetin nanoparticles (1 μM), the production of these cytokines was assessed using ELISA kits (DuoSet ELISA Development kit, cat No. DY210-05) according to the manufacturer protocol.

Moreover, cellular reactive oxygen species (ROS) levels of simulated hDPSCs were evaluated by the DCFDA-ROS Detection Assay Kit (ab113851). The assay uses the cell-permeant reagent 2', 7'-dichlorofluorescein diacetate (DCFDA), a fluorogenic dye that assesses hydroxyl, peroxyl, and other ROS activities within the cells. After that, DCFDA is deacetylated by cellular esterases to a non-fluorescent compound, which is later oxidized by ROS into 2', 7'-dichlorofluorescein (DCF). Intracellular ROS production was respectively detected by measuring the fluorescence intensity of cells with a microplate reader with excitation of 490 nm and emission of 530 nm wavelengths as described previously^46^. The measured values of fluorescence were demonstrated as fluorescence intensity/mg protein.

As the immune cell are one of the critical components in the inflammatory reactions, same tests were repeated on inflamed monocytes. These cells were purchased from Sara Research Co. as U937 cell line.

### Mineralization induction of hDPSCs by hesperetin nanocrystals

To evaluate the mineralization induction capacity of hesperetin and hesperetin nanocrystals on hDPSCs, these cells were seeded with the number of 5 × 10^5^, and the culture medium containing 1 μM hesperetin and nano-hesperetin was used for feeding these cells. The culture medium was replaced every three days. After 21 days, the Alizarin red staining was performed as mentioned previously^47^ to measure the induction of mineralization in hDPSCs. Briefly, the wells were washed with PBS (three times), fixed with 4% formaldehyde for 20 min, and then washed with deionized water for 10 min. Cells were stained with 1% ARS and shook for 15 min. To quantify the results, the ARS solution was extracted by adding a 10% acetic acid solution for 30 min with constant shaking and then neutralized with a 10% ammonium hydroxide solution, which was followed by colorimetric detection at 405 nm using an ELISA reader (Bio Teck, Germany).

### Expression of osteogenic genes and proteins in hDPSCs stimulated by hesperetin nanocrystals

For detecting osteogenic marker expression in the presence of hesperetin and hesperetin nanocrystals, 1 × 10^6^ hDPSCs were cultured in each well of 6 well-plates containing osteogenic medium which included *α*-MEM medium supplemented with 100 IU/ml penicillin, 100 μg/ml streptomycin, 10 nM dexamethasone and 0.2 mM sodium L-ascorbyl-2-phosphate, 10 mM *β*-glycerol phosphate, and 10% FBS. Three wells were treated with 1 μM hesperetin, three wells were treated with 1 μM HS nanoparticles, and three wells remained untreated as the control group. After 21 days, total RNA was extracted from each sample according to our previous study^48^. Briefly, the wells were trypsinized, and after centrifuging a 1 mL TRIzol reagent (Cat No: 15596-026, Invitrogen, USA) was added, and then 200 μL chloroform (Merck, Darmstadt, Germany) was added and centrifuged at 4 °C at 12,000 rpm for 20 min. The upper phase containing RNA was mixed with isopropanol (Sigma-Aldrich) and incubated at 4 °C for 15 min and centrifuged at 4 °C at 12,000 rpm for 20 min. Then the supernatant was discarded, and 1 mL ethanol (75% v/v) was added to dissolve the pellet and centrifuged at 4 °C at 7500 rpm for 10 min. Then the supernatant was discarded and 20 μL water was added. The concentration of total RNA was measured with a Nanodrop system (Thermo Scientific). RNA solutions were incubated with the DNase1 kit (Cat No: en0521, Fermentaz). Then, 1 μg of total RNA was used for cDNA synthesizing by a cDNA synthesis kit (Cat No: YT4500). The expression level of five osteogenic genes was evaluated by specific primers mentioned in Table 1. The GAPDH gene was used as a housekeeping gene for normalization. The expression was measured by an RT-PCR system, including Rotor-Gene Corbett System 6000. The experiment was conducted in triplicate, and the data were analyzed by a convenient Pfaffl method.

**Table 1:** Sequences and melting temperature of primers.

Target	Sense and antisense sequences 5′–3ʹ	tA (°C)
COL1A1	F: CAAGAGGAAGGCCAAGTCGAG R: AGATCACGTCATCGCACAACA	59
OCN	F: TGTGTGAGCTCAATCCGGACT R: CCTGGAGAGGAGCAGAACTGG	61
RUNX-2	F: CTCACTGCCTCTCACTTGCC R: CTGTACACACATCTCCTCCC	65
ALP	F: AGAGTCACTCCTGCCTTCAC R: GTGTCAACAGGATCCAGGCAT	61
BSP	F: CACCAGTACCAACAGCACAGA R: GCATTGGCTCCAGTGACACTT	61
GAPDH	F: GAAGGTGAAGGTCGGAGTC R: GAAGATGGTGATGGGATTTC	65

Western blot analysis was conducted to evaluate the expression of osteogenic proteins in hDPSCs treated with hesperetin and hesperetin nanocrystals. The samples were prepared as well as real-time PCR samples. After 21 days, the cells were collected and lysed in ice-cold cell lysis buffer solution (NaCl, NP-40, and Tris–HCl), including cocktail enzyme inhibitors. Then, the solutions were sonicated and centrifuged at 14,000*g* for 20 min. The total protein contents were measured in the supernatant by the Picodrop spectrophotometer system (Model No: PICOPET01, Serial No. 000212/1) and resolved by the SDS-PAGE method. The samples were incubated overnight at 4 °C in the following primary antibody solutions including Alkaline phosphatase (ALP, Cat No: ab39256, Abcam company), collagen type Ι alpha Ι (COL1α1, Cat No: sc-517593, Santa Cruz Biotechnology, Inc.), osteocalcin (OCN, Cat No: sc-293414, Santa Cruz Biotechnology, Inc.), Bone Sialoproteins II (BSP, Cat No: sc-73634, Santa Cruz Biotechnology, Inc.), Runt-related transcription factor (RUNX2, Cat No: sc-390351, Santa Cruz Biotechnology, Inc.), and β-Actin (Cat No: sc-47778, Santa Cruz Biotechnology, Inc.). After that, the samples were incubated with secondary HRP-conjugated anti-IgG antibody (Cat No: sc-2357, Santa Cruz Biotechnology, Inc.) for 1 h at room temperature. The immunoreactive blots were detected by the ECL plus solution kit (BioRad) according to the manufacturer’s instruction to visualize the reactive proteins on the blots. This experiment was performed in triplicate.

### Statistical analysis

The data were determined as the mean ± standard deviation and comprised by using One-way ANOVA and Tukey test analysis with Prism software (version 8.0, GraphPad, San Diego, CA, USA). *p*-value < 0.05 was considered statistically significant (Supplementary information S1).

### Ethics approval

All experimental protocols were approved by the Ethics committee of Tabriz University of Medical Sciences (TUMS), which complied with the Helsinki declaration (Approval No. IR.TBZMED.REC.1398.475).

## Supplementary Information

Supplementary Figures.

## Data Availability

All data generated and/or analyzed during this study are included in this published article. The datasets used and/or analyzed during the current study are available from the corresponding author on reasonable request.
