# Supplementary material for: Synthesis, characterization, and evaluation of Hesperetin nanocrystals for regenerative dentistry
Source: Sci Rep. 2023 Feb 6;13:2076. doi: 10.1038/s41598-023-28267-y (PMC9902453; doi:10.1038/s41598-023-28267-y)
Supplement: Supplementary file 1 — Supplementary Figures. [file 41598_2023_28267_MOESM1_ESM.pdf]

SUPPLEMENTARY MATERIAL

Title: Synthesis, characterization, and evaluation of Hesperetin nanocrystals for regenerative dentistry

Mahdieh Alipour, DDS [1], Simin Sharifi, Ph.D.[1], Mohammad Samiei, DDS, MSc [2], Shahriar Shahi, DDS, MSc [1], Marziyeh Aghazadeh, DDS, MSc[3, 4*], Solmaz Maleki Dizaj, Ph.D.[1*].

[1] Dental and Periodontal Research Center, Faculty of Dentistry, Tabriz University of Medical Sciences, Tabriz, Iran.

[2] Department of Endodontics, Faculty of Dentistry, Tabriz University of Medical Sciences, Tabriz, Iran.

[3] Stem Cell Research Center, Tabriz University of Medical Sciences, Tabriz, Iran

[4] Department of Oral Medicine, Faculty of Dentistry, Tabriz University of Medical Sciences, Tabriz, Iran

[*] Corresponding authors:

Solmaz Maleki Dizaj:

E-mail: maleki.s.89@gmail.com

Address: Daneshgah St, Golgasht St, Faculty of Dentistry, [1]Dental and Periodontal Research Center, Tabriz University of Medical Sciences, Tabriz, Iran.

Phone number: +9841- 33353161

Marziyeh Aghazadeh:

E-mail: maghazadehbio@gmail.com

Address: Daneshgah St, Golgasht St, Faculty of Dentistry, Tabriz University of Medical Sciences, Tabriz, Iran.

Phone number: +9841- 33355965

A)

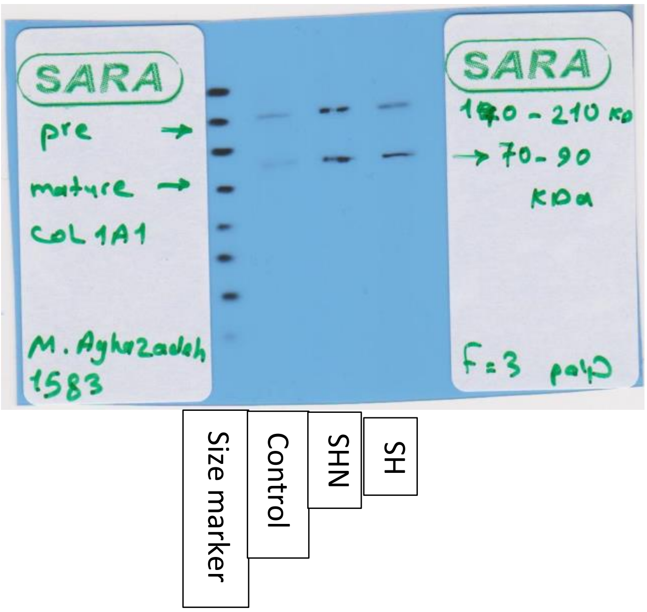

B)

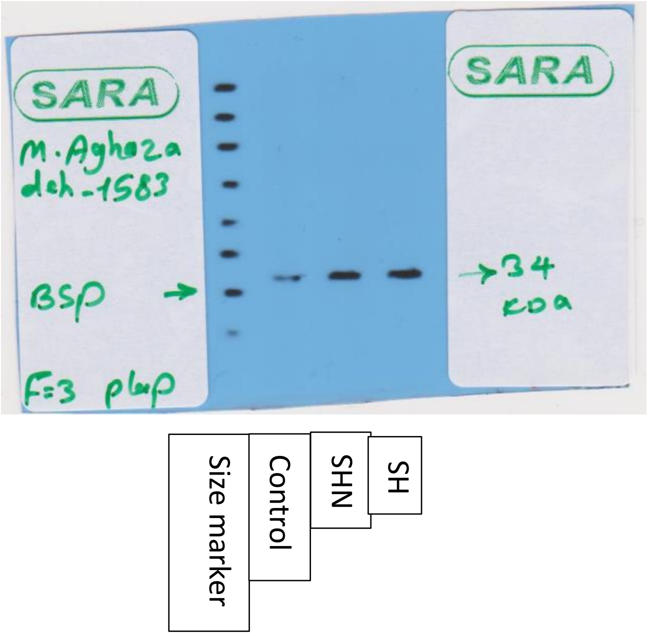

C)

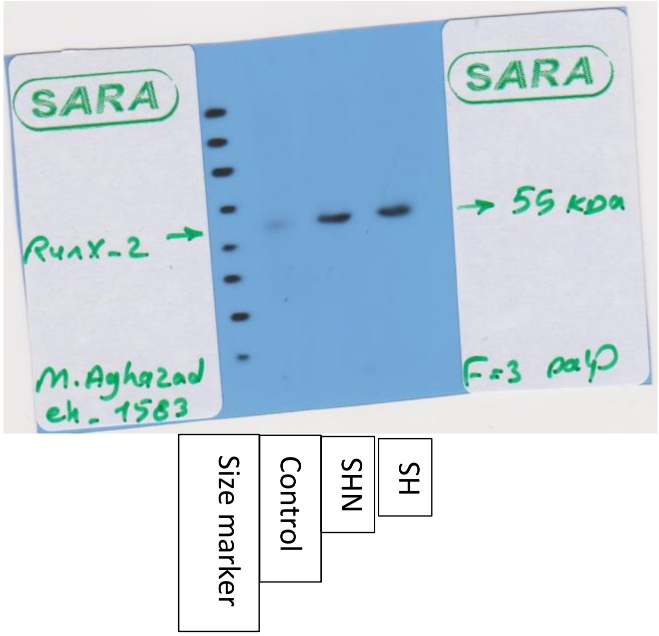
SARA
SARA
Runx-2
55 kDa
M.Aghazadeh_1583
Size marker
Control
SHN
SH

D)

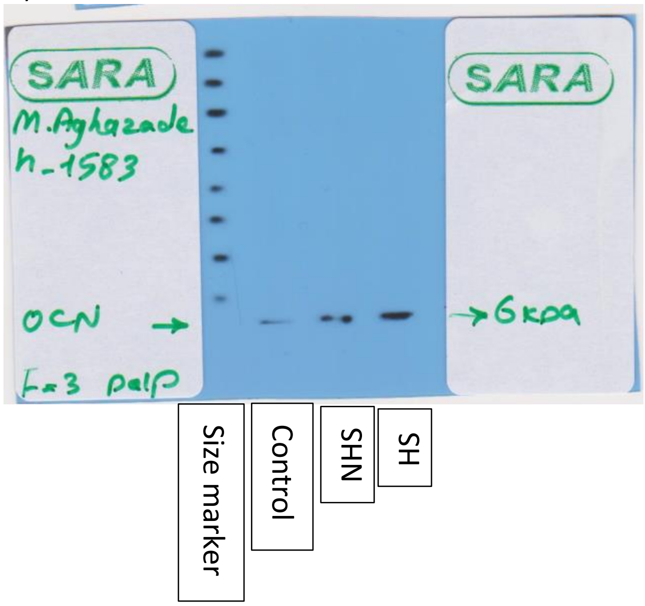
SARA
SARA
M.Aghazadeh_1583
OCN
6 kDa
Size marker
Control
SHN
SH

E)

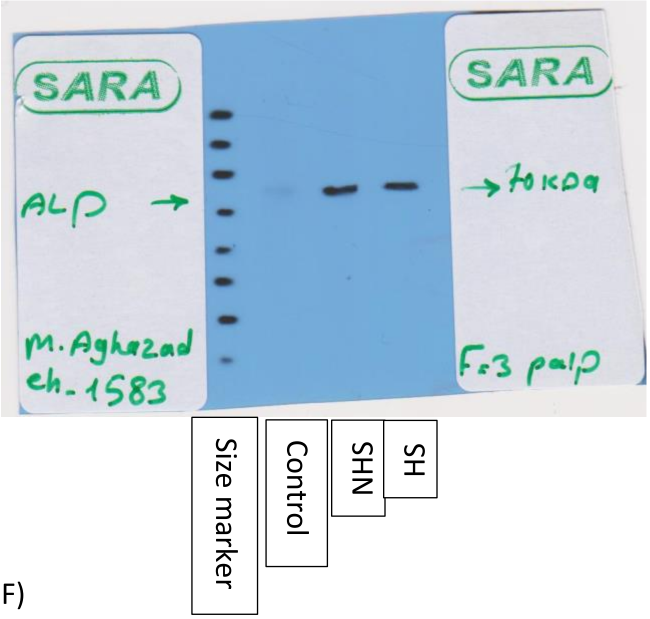

F)

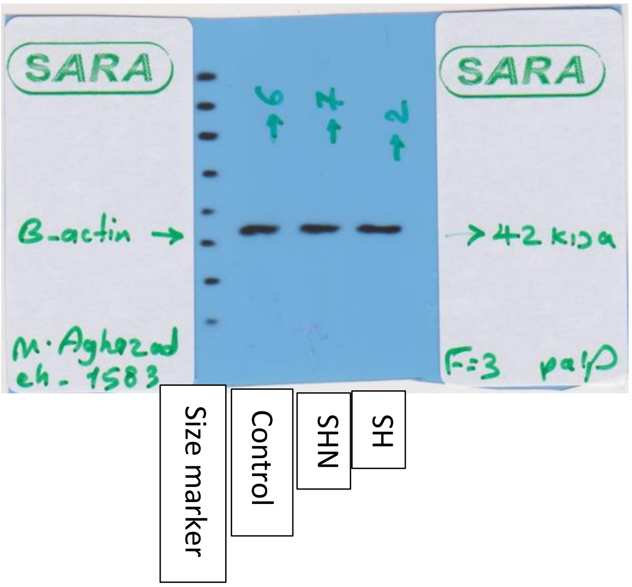

Figure S1. Osteogenic differentiation of hDPSCs cultured in presence of Hesperetin (HS) and Hesperetin nanoparticles (NHS). The human dental pulp stem cells are considered as a control group. The cell lysates were prepared and used for western blot with A) Col1a1, B) BSP, C) RUNX2, D) OCN, E) ALP, F) B-actin. The gels were not cropped.
